# Risk Behaviors and Risk Factors for HIV Infection among Participants in the Bangkok Tenofovir Study, an HIV Pre-Exposure Prophylaxis Trial among People Who Inject Drugs

**DOI:** 10.1371/journal.pone.0092809

**Published:** 2014-03-25

**Authors:** Michael Martin, Suphak Vanichseni, Pravan Suntharasamai, Udomsak Sangkum, Philip A. Mock, Manoj Leethochawalit, Sithisat Chiamwongpaet, Roman J. Gvetadze, Somyot Kittimunkong, Marcel E. Curlin, Dararat Worrajittanon, Janet M. McNicholl, Lynn A. Paxton, Kachit Choopanya, for the Bangkok Tenofovir Study Group

**Affiliations:** 1 Thailand MOPH – U.S. CDC Collaboration, Nonthaburi, Thailand; 2 Centers for Disease Control and Prevention, Atlanta, Georgia, United States of America; 3 Bangkok Tenofovir Study Group, Bangkok, Thailand; 4 Bangkok Metropolitan Administration, Bangkok, Thailand; 5 Thailand Ministry of Public Health, Nonthaburi, Thailand; Fundacion Huesped, Argentina

## Abstract

**Introduction:**

HIV spread rapidly among people who inject drugs in Bangkok in the late 1980s. In recent years, changes in drug use and HIV-associated risk behaviors have been reported. We examined data from the Bangkok Tenofovir Study, an HIV pre-exposure prophylaxis trial conducted among people who inject drugs, to assess participant risk behavior and drug use, and to identify risk factors for HIV infection.

**Methods:**

The Bangkok Tenofovir Study was a randomized, double-blind, placebo-controlled trial. HIV status was assessed monthly and risk behavior every 3 months. We used generalized estimating equations logistic regression to model trends of injecting, needle sharing, drugs injected, incarceration, and sexual activity reported at follow-up visits; and proportional hazards models to evaluate demographic characteristics, sexual activities, incarceration, drug injection practices, and drugs injected during follow-up as predictors of HIV infection.

**Results:**

The proportion of participants injecting drugs, sharing needles, and reporting sex with more than one partner declined during follow-up (p<0.001). Among participants who reported injecting at enrollment, 801 (53.2%) injected methamphetamine, 559 (37.1%) midazolam, and 527 (35.0%) heroin. In multivariable analysis, young age (i.e., 20–29 years) (p = 0.02), sharing needles (p<0.001), and incarceration in prison (p = 0.002) were associated with incident HIV infection. Participants reporting sex with an opposite sex partner, live-in partner, casual partner, or men reporting sex with male partners were not at a significantly higher risk of HIV infection compared to those who did not report these behaviors.

**Conclusion:**

Reports of HIV-associated risk behavior declined significantly during the trial. Young age, needle sharing, and incarceration were independently associated with HIV infection. Sexual activity was not associated with HIV infection, suggesting that the reduction in HIV incidence among participants taking daily oral tenofovir compared to those taking placebo was due to a decrease in parenteral HIV transmission.

## Introduction

HIV spread rapidly among people who inject drugs (PWID) in Bangkok in the late 1980s [1] and HIV prevalence has remained high, ranging from 30% to 50% through 2009, the highest among risk populations surveyed [2]. During 2005–2012, we conducted the Bangkok Tenofovir Study (BTS), a randomized, double-blind, placebo-controlled trial, that showed that daily prophylaxis with tenofovir disoproxil fumarate (tenofovir) could reduce the risk of HIV infection among PWID [3], [4]. The study provided an opportunity to determine whether trial participation would lead to increased risk behavior, to assess drug use, and to examine the relationship between injection practices, sexual activities, and incident HIV infection.

Risk factor analysis also helps interpret BTS results: if participants were at risk of HIV infection because of drug injection practices, the efficacy result suggests that tenofovir reduces parenteral HIV transmission; if, on the other hand, HIV infection was primarily related to sexual exposure, the efficacy result lends support to trials showing pre-exposure prophylaxis can reduce sexual transmission [5], [6], [7], but the impact on parenteral transmission remains unclear.

In this manuscript, we describe HIV-associated risk behaviors and drug use reported by participants, and examine risk behaviors and drugs injected to determine predictors of HIV infection.

## Methods

Descriptions of community engagement, enrollment, and safety and efficacy results have been published [3], [4]. Ethical Review Committees of the Bangkok Metropolitan Administration (BMA) and the Thailand Ministry of Public Health and the U.S. Centers for Disease Control and Prevention Institutional Review Board approved the study protocol and consent forms. The study was conducted in 17 BMA drug-treatment clinics according to the principles expressed in the Declaration of Helsinki. HIV-uninfected individuals aged 20 to 60 years who reported injecting drugs during the previous year were candidates for the study. Volunteers meeting all eligibility criteria could enroll after providing written informed consent. A total of 2413 people enrolled and were randomly assigned in a 1:1 ratio to receive daily oral tenofovir 300 mg or placebo.

At enrollment and monthly (28 days) visits, individualized risk-reduction counseling was provided and oral fluid was tested for HIV antibodies (OraSure Technologies Inc., Bethlehem, USA). Newly infected individuals were referred for care according to national guidelines [8]. Risk behavior was assessed at enrollment, 3-monthly, and when oral fluid HIV test were reactive using an audio computer-assisted self-interview (ACASI). When a participant was incarcerated, staff contacted prison authorities to arrange a study visit. Visits were not conducted in police cells (i.e., jails) because of the unpredictable occurrence and relatively short duration of incarceration. Thai law prohibits the distribution of needles to inject illicit drugs and needles were not provided to participants; however, sterile needles are available without a prescription at low cost (5–10 baht/0.12–0.25 US dollars) in pharmacies in Bangkok.

Generalized estimating equations logistic regression [9] was used to model trends of injecting, sharing, incarceration, and sexual activity. HIV incidence and exact 95% Poisson confidence intervals (CI) were calculated per 1000 person-years of HIV-negative observation. We used proportional hazards models [10] to evaluate baseline demographic characteristics; and, incarceration, drug injection, and sexual activity reported the visit of the first positive HIV test as predictors of HIV infection, controlling for treatment group in all models. Variables associated with HIV infection in bivariate analysis (p<0.1) were evaluated in a multivariable model. We used SAS (Version 9; SAS Institute, Cary, USA) for analysis.

## Results

We described trial results in previous publications [3], [4]. Briefly, from June 2005 through July 2010, we screened 4094 volunteers; 2413 (58.9%) were deemed eligible and enrolled. Their median age was 31 years (range 20–59) and 1924 (79.7%) were male. Participants were followed for 9665 person-years (mean 4.0 years; maximum 6.9 years). Fifty participants became infected during follow-up: 17 in the tenofovir group (incidence, 0.35 per 100 person-years) and 33 in the placebo group (incidence, 0.68 per 100 person-years) indicating a 48.9% reduction in HIV incidence (95% CI, 9.6 to 72.2; p = 0.01) among participants randomized to tenofovir.

All participants reported injecting drugs during the year before enrollment. The number reporting injection drug use during the previous 3 months decreased from 1507 (62.7%) at enrollment, to 426 (22.7%) at month 12, and to 117 (17.5%) at month 72 (p<0.001) (Table 1). Similarly, reports of sharing needles decreased from 435 (18.1%) at enrollment, to 44 (2.4%) at month 12, and to 8 (1.2%) at month 72 (p<0.001). Excluding enrollment, 1014 (42.0%) participants reported injecting drugs during the study; 35 (70.0%) of those who became HIV-infected and 979 (41.5%) of those who remained HIV-uninfected.

**Table 1:** Risk behaviors reported by participants in the Bangkok Tenofovir Study, Thailand, 2005–2012^a^.

	Study Visit								Reported at least once Months 3 to 72	
	Enrollment	Month 12	Month 24	Month 36	Month 48	Month 60	Month 72		HIV infected^b^	HIV uninfected
	n = 2405no. (%)	n = 1876no. (%)	n = 1712no. (%)	n = 1540no. (%)	n = 1328no. (%)	n = 1032no. (%)	n = 667no. (%)	P value	n = 50no. (%)	n = 2361no. (%)
**Drug injection practices in past 3 months**										
Injected drugs	1507 (62.7)	426 (22.7)	322 (18.8)	270 (17.5)	221 (16.6)	166 (16.1)	117 (17.5)	<0.001	35 (70.0)	979 (41.5)
Shared needles	435 (18.1)	44 (2.4)	40 (2.3)	31 (2.0)	29 (2.2)	14 (1.4)	8 (1.2)	<0.001	14 (28.0)	291 (12.3)
Injected daily	204 (8.5)	98 (5.2)	87 (5.1)	86 (5.6)	64 (4.8)	57 (5.5)	47 (7.1)	0.43	9 (18.0)	363 (15.4)
**Drugs injected in past 3 months**										
Methamphetamine	801 (33.3)	139 (7.4)	117 (6.8)	110 (7.1)	78 (5.9)	68 (6.6)	39 (5.9)	<0.001	21 (42.0)	627 (26.6)
Midazolam	559 (23.2)	259 (13.8)	192 (11.2)	168 (10.9)	135 (10.2)	108 (10.5)	75 (11.2)	<0.001	17 (34.0)	580 (24.6)
Heroin	527 (21.9)	161 (8.6)	103 (6.0)	86 (5.6)	73 (5.5)	67 (6.5)	42 (6.3)	<0.001	14 (28.0)	501 (21.2)
**Drug or drug combinations injected in past 3 months**										
Methamphetamine only	448 (18.6)	42 (2.2)	43 (2.5)	40 (2.6)	27 (2.0)	18 (1.7)	13 (2.0)	<0.001	11 (22.0)	338 (14.3)
Midazolam only	203 (8.4)	104 (5.5)	79 (4.6)	69 (4.5)	51 (3.8)	38 (3.7)	28 (4.2)	<0.001	4 (8.0)	400 (16.9)
Heroin only	204 (8.5)	54 (2.9)	35 (2.0)	20 (1.3)	19 (1.4)	16 (1.6)	6 (0.9)	<0.001	8 (16.0)	244 (10.3)
Methamphetamine and midazolam only	117 (4.9)	41 (2.2)	33 (1.9)	30 (2.0)	15 (1.1)	16 (1.6)	8 (1.2)	<0.001	15 (30.0)	670 (28.4)
Methamphetamine and heroin only	105 (4.4)	15 (0.8)	11 (0.6)	7 (0.5)	6 (0.5)	8 (0.8)	4 (0.6)	<0.001	18 (36.0)	544 (23.0)
Midazolam and heroin only	102 (4.2)	57 (3.0)	29 (1.7)	30 (2.0)	25 (1.9)	18 (1.7)	16 (2.4)	<0.001	10 (20.0)	557 (23.6)
Methamphetamine, midazolam, and heroin only	54 (2.3)	21 (1.1)	16 (0.9)	21 (1.4)	16 (1.2)	17 (1.7)	7 (1.1)	0.76	20 (40.0)	807 (34.2)
Other drugs or drug combinations	274 (11.4)	92 (4.9)	77 (4.5)	53 (3.4)	62 (4.7)	35 (3.4)	35 (5.3)	<0.001	11 (21.2)	356 (15.1)
**Incarceration in past 3 months**										
In jail	552 (23.0)	243 (12.9)	208 (12.1)	188 (12.2)	128 (9.6)	101 (9.8)	53 (8.0)	<0.001	31 (62.0)	1070 (45.3)
In prison	389 (16.2)	244 (13.0)	223 (13.0)	215 (14.0)	153 (11.5)	117 (11.3)	60 (9.0)	0.04	29 (58.0)	964 (40.8)
**Sexual activity in past 3 months**										
More than one sexual partner	522 (21.7)	207 (11.0)	188 (11.0)	139 (9.0)	113 (8.5)	82 (7.9)	43 (6.5)	<0.001	19 (38.0)	837 (35.4)
Sex with casual partner	914 (38.0)	428 (22.8)	349 (20.4)	281 (18.2)	209 (15.7)	160 (15.5)	97 (14.5)	<0.001	32 (64.0)	1414 (59.8)
Men reporting sex with male partner	91/1913 (4.8)	36/1487 (2.4)	25/1356 (1.8)	16/1224 (1.3)	16/1054 (1.5)	9/820 (1.1)	5/520 (1.0)	<0.001	8 (16.0)	196 (8.3)

a Risk behaviors reported at annual visits are shown in the table; data from all 3-monthly visits through month 72 were used in the generalized estimating equations logistic regression analysis.

b Data from two participants found to be HIV-infected at enrollment are excluded.

Among participants who reported injecting at enrollment, 801 (53.2%) injected methamphetamine, 559 (37.1%) midazolam, and 527 (35.0%) heroin. Reports of injecting declined for all drugs during follow-up, but among injectors, the proportion injecting midazolam increased to 64.1% and methamphetamine declined to 33.3% at month 72.

At enrollment, 1922 (79.7%) participants reported having been incarcerated in the past; 1905 (79.0%) in jail and 1403 (58.1%) in prison. Reports of incarceration in jail in the previous 3 months declined from 552 (23.0%) at enrollment to 53 (8.0%) at month 72 (p<0.001) and in prison from 389 (16.2%) at enrollment to 60 (9.0%) at month 72 (p = 0.04) (Table 1). During the study, 117 (4.9%) participants reported injection drug use while incarcerated, 58 (2.4%) in jail and 66 (2.7%) in prison; 104 (4.3%) reported sex while incarcerated, 58 (2.4%) in jail and 71 (3.0%) in prison.

At enrollment, 522 (21.7%) participants reported sex with more than one partner during the previous 3 months and this decreased to 43 (6.5%) at month 72 (p<0.001). Sex with casual partners declined from 914 (38.0%) at enrollment to 97 (14.5%) at month 72 (p<0.001). At enrollment, 91 (4.8%) of the 1913 men reported sex with a male partner in the previous 3 months; 73 (80.2%) of these men also reported sex with a female partner. The number of men reporting sex with men declined to 5 (1.0%) at month 72 (p<0.001). A total of 1044 (43.4%) participants reported sex with a live-in partner during the 3 months before enrollment, and only 78 (7.5%) reported 100% condom use with this partner; 914 (38.0%) reported sex with a casual partner, and 304 (37.4%) of the 813 male participants reported 100% condom use. A data collection problem limited complete assessment of condom use with casual partners to men.

In bivariate analysis, we found that participants 20 to 29 years old were more likely to become HIV infected than older participants (hazard ratio [HR] 2.0; 95% CI, 1.1–3.5; p = 0.02) and that participants who reported injecting drugs (HR 1.9; 95% CI, 1.0–3.5; p = 0.04) (not shown), sharing needles (HR 9.6; 95% CI, 4.5–20.7; p<0.001), or being in jail (HR 3.1; 95% CI, 1.6–5.7; p<0.001) or prison (HR 3.1; 95% CI, 1.7–5.6; p<0.001) were more likely to become HIV infected than those who did not report these risk factors (Table 2). Participants reporting sex with a partner of the opposite sex (p = 0.79), sex with a live-in partner (p = 0.11), sex with a casual partner (p = 0.13), or men reporting sex with male partners (0.50) were not at a higher risk of HIV infection compared to those who did not report these behaviors. Participants who reported unprotected sex with a live-in partner were less likely to become HIV-infected than participants who did not report unprotected sex with a live-in partner (HR 0.4; 95% CI, 0.2–0.9; p = 0.02). Among men, unprotected sex with a casual partner was not associated with incident HIV infection (HR 1.7; 95% CI, 0.7–4.1; p = 0.23). Among women, condom use data were available on 12 (26.1%) women who reported having sex with a casual partner in the previous 3 months at the month 12 visit, 18 (36.0%) women at month 24, 33 (89.2%) at month 36, and 100% at month 48 and thereafter. We analyzed the data that were available from women reporting sex with a casual partner and did not find an association between unprotected sex and incident HIV infection (HR 1.9; 95% CI, 0.2–15.3; p = 0.55). The small number of women with incident HIV-infection (n = 11) limits the power of the analysis.

**Table 2:** Bivariate and multivariable analysis, controlling for treatment group, of demographic characteristics at enrollment and risks behaviors reported during the 3 months before HIV infection among participants in the Bangkok Tenofovir Study, 2005–2012.

Characteristics and risks	HIV-infected	Person-years	HIV incidence^a^ (95% CI)	Bivariate analysis			Multivariable analysis	
				Hazard Ratio (95% CI)	P value	Trend P value	Hazard Ratio (95% CI)	P value
Sex								
Male	39	6745	5.8 (4.1–7.9)	1.0				
Female	11	1770	6.2 (3.1–11.1)	1.1 (0.6–2.1)	0.80		Not included	
Age (years) at enrollment								
≥30	22	5208	4.2 (2.7–6.4)	1.0			1.0	
20–29	28	3307	8.5 (5.6–12.2)	2.0 (1.1–3.5)	0.02		1.9 (1.1–3.4)	0.02
Education								
Secondary or more	22	4347	5.1 (3.2–7.7)	1.0				
Primary or less	28	4168	6.7 (4.5–9.7)	1.3 (0.8–2.3)	0.30		Not included	
In methadone program in past 3 months								
No	40	6517	6.1 (4.4–8.4)	1.0				
Yes	10	1998	5.0 (2.4–9.2)	0.8 (0.4–1.7)	0.62		Not included	
Injection frequency in past 3 months								
Did not inject	34	6809	5.0 (3.5–7.0)	1.0				
Weekly or less	11	1218	9.0 (4.5–16.2)	1.8 (0.9–3.6)				
Daily	5	489	10.2 (3.3–23.9)	2.0 (0.8–5.2)	0.11	0.04	Not significant	
Injected heroin in past 3 months								
No	45	7924	5.7 (4.1–7.6)	1.0				
Yes	5	591	8.5 (2.8–19.7)	1.5 (0.6–3.7)	0.41		Not included	
Injected methamphetamine in past 3 months								
No	43	7888	5.5 (4.0–7.3)	1.0				
Yes	7	628	11.2 (4.5–23.0)	2.1 (0.9–4.6)	0.08		Not significant	
Injected midazolam in past 3 months								
No	45	7499	6.0 (4.4–8.0)	1.0				
Yes	5	1016	4.9 (1.6–11.5)	0.9 (0.3–2.0)	0.65		Not included	
Shared needles in past 3 months								
No	42	8332	5.0 (3.6–6.8)	1.0			1.0	
Yes	8	183	43.8 (18.9–86.2)	9.6 (4.5–20.7)	<0.001		8.9 (4.1–19.3)	<0.001
In police cell (jail) in past 3 months								
No	36	7553	4.8 (3.3–6.6)	1.0				
Yes	14	962	14.6 (8.0–24.4)	3.1 (1.6–5.7)	<0.001		Not significant	
In prison in past 3 months								
No	35	7489	4.7 (3.3–6.5)	1.0			1.0	
Yes	15	1026	14.6 (8.2–24.1)	3.1 (1.7–5.6)	<0.001		2.7 (1.4–4.9)	0.002
Sex with opposite sex partner in past 3 months								
No	22	3846	5.7 (3.6–8.7)	1.0				
Yes	28	4669	6.0 (4.0–8.7)	1.1 (0.6–1.9)	0.79		Not included	
Sex with live-in partner in past 3 months								
No	31	4295	7.2 (4.9–10.3)	1.0				
Yes	19	4220	4.5 (2.7–7.0)	0.6 (0.4–1.1)	0.11		Not included	
Sex with casual partner in past 3 months								
No	36	6814	5.3 (3.7–7.3)	1.0				
Yes	14	1701	8.2 (4.5–13.8)	1.6 (0.9–3.0)	0.13		Not included	
Men reporting sex with men								
No	39	6633	5.9 (4.2–8.0)	1.0				
Yes	0	112	0.0	0.0 (0.0–5.9)	0.50		Not included	

a HIV incidence per 1000 person-years. CI = confidence interval.

Multivariable analysis showed that younger age (i.e., 20 to 29 years old) (HR 1.9; 95% CI, 1.1–3.4; p = 0.02), sharing needles (HR 8.9; 95% CI, 4.1–19.3; p<0.001), and incarceration in prison (HR 2.7; 95% CI, 1.4–4.9; p = 0.002) were independently associated with incident HIV infection (Table 2).

## Discussion

Participant reports of injecting drugs, sharing needles, and multiple sex partners declined during follow-up. This may be due to HIV preventive services and monthly HIV testing provided as part of the study. Multivariable analysis showed that young age, sharing needles, and incarceration were independent predictors of incident HIV infection. Reported sexual activity was modest; 1883 (78.3%) participants reported zero or one sexual partner during the 3 months before enrollment and, consistent with previous studies among PWID in Bangkok, sexual activity was not associated with HIV infection [11], [12]. Participants who reported unprotected sex with live-in partners were at a lower risk of HIV infection than other participants. Although this is counterintuitive, a previous study among PWID in the same clinics [11] found that participants who reported sex were less likely to become HIV-infected than those who did not report having sex, suggesting that PWID who have a sexual partner or a live-in partner are less likely to engage in HIV-associated risk behaviors. HIV transmission in Thailand is concentrated among PWID, men who have sex with men (MSM), and sex workers; accounting for 55% of new infections in 2010 [13]. Some participants may have been exposed to HIV through sex, but these results, and the efficiency of parenteral HIV transmission (i.e., five to 10 times as great as heterosexual transmission) [14], suggest that sharing needles was the primary mode of HIV transmission among BTS participants, and that the efficacy result was due to a reduction in parenteral HIV transmission among tenofovir recipients.

Studies among PWID [11] and MSM [15] in Thailand have found higher HIV incidence rates among younger participants and investigators suggest the higher rates are due to increased HIV associated risk behaviors among these young participants. We controlled for injecting and sexual risk behaviors in our analysis, but it is possible that younger participants underreported risk behaviors. In addition, we found better study drug adherence among participants aged 40 years and older than among younger participants [3].

Our results add to existing evidence demonstrating an association between incarceration and HIV infection [12], [16], [17], [18], [19], [20], [21], [22], [23], [24]. It is important to note that this association has been reported in many countries and is not unique to Thailand [16], [17], [18], [19], [20], [21], [22]. Despite this evidence and recommendations that drug treatment and HIV prevention tools be made available to incarcerated drug users, these services remain rare in prison [22], [25].

The HIV incidence among placebo recipients in the study was 0.7 per 100 person-years compared to 5.8 per 100 person-years in a preparatory trial 1995–1999 [11] and 3.4 per 100 person-years during the 1999–2003 AIDSVAX B/E HIV vaccine trial [26] conducted among PWID in the same clinics. This decline in HIV incidence is likely due to multiple factors, including HIV prevention services offered in the drug-treatment clinics, decreases in the frequency of drug injection and needle sharing, and monthly HIV counseling and testing provided in the study. Despite this decline, the HIV incidence among PWID in this study is more than 20 times the estimated incidence among adults in Thailand (0.03% in 2011) [13] and more than twice as high as placebo recipients in the RV144 HIV vaccine trial (0.3 per 100 person-years); 4151 (52.0%) of whom reported they were at medium to high risk of HIV infection [27].

The study has a number of limitations. A data collection problem prevented some women from reporting condom use with casual partners. Participants were willing to come to clinics monthly and report injection practices and sexual activity. Their risk behaviors may differ from PWID not in the study, limiting the generalizability of the results. However, using respondent-driven sampling, investigators estimated there were 3595 people injecting drugs in Bangkok in 2004, [28] and 4200 in 2009 [29], suggesting a substantial proportion of PWID enrolled in the BTS allowing generalization of the results to PWID in Bangkok. Participants may have under-reported stigmatized and illegal behaviors [30], but the illegality and stigma attached to these activities did not change during the trial; so, rates of under-reporting should have remained constant, allowing comparisons over time.

After an explosive outbreak of HIV among PWID in Bangkok in the late 1980s [1], new infections continued to occur at a rate that maintained HIV prevalence at 30% to 50% through 2009 [2]. But now, with declining numbers of PWID [28], [31], decreases in the frequency of injecting and sharing, and the demonstration that pre-exposure prophylaxis can reduce parenteral HIV transmission [3], it may be possible to end the HIV epidemic among PWID in Bangkok. The World Health Organization has published guidance assessing interventions likely to have the greatest impact on HIV prevention among PWID [32]. Based on our data, pre-exposure prophylaxis should be considered among these interventions, and the public health community should work to make a package of effective HIV prevention tools available to PWID, specifically to young PWID who report sharing needles and to incarcerated PWID.
